# Impact of Combined Processes Involving Ultrasound and Pulsed Electric Fields on ENNs, and OTA Mitigation of an Orange Juice-Milk Based Beverage

**DOI:** 10.3390/foods12081582

**Published:** 2023-04-08

**Authors:** Albert Sebastià, Mara Calleja-Gómez, Noelia Pallarés, Francisco J. Barba, Houda Berrada, Emilia Ferrer

**Affiliations:** Preventive Medicine and Public Health, Food Science, Toxicology and Forensic Medicine Department, Faculty of Pharmacy, Universitat de València, Avda. Vicent Andrés Estellés, 46100 Burjassot, Spain

**Keywords:** enniatins, Ochratoxin A, pulsed electric field, ultrasounds, mitigation

## Abstract

In recent years, several innovative food processing technologies such as ultrasound (USN) and pulsed electric fields (PEF) have emerged in the market, showing a great potential both alone and in combination for the preservation of fresh and processed products. Recently, these technologies have also shown promising applications to reduce mycotoxin levels in food products. Therefore, the objective of this study is to investigate the potential of the combined treatments USN + PEF and PEF + USN on the reduction of Ochratoxin A (OTA) and Enniatins (ENNs) of an orange juice mixed with milk beverage. For this purpose, the beverages were elaborated in the laboratory and individually spiked with mycotoxins at a concentration of 100 µg/L. They were then treated by PEF (30 kV, 500 kJ/Kg) and USN (20 kHz, 100 W, at a maximum power for 30 min). Finally, mycotoxins were extracted using dispersive liquid-liquid microextraction (DLLME), and liquid chromatography coupled to tandem mass spectrometry (LC-MS/MS-IT) was employed to determine them. The results showed promising applications, with reductions up to 50% for OTA and up to 47% for Enniatin B (ENNB) after the PEF + USN treatment combination. Lower reduction rates, up to 37%, were obtained with the USN + PEF combination. In conclusion, the combination of USN and PEF technologies could be a useful tool to reduce mycotoxins in fruit juices mixed with milk.

## 1. Introduction

In recent times, an increasing demand for minimally processed fresh fruits and vegetable products has been observed worldwide due to healthy lifestyle recommendations. These products present a high nutritional quality and contribute to a well-balanced diet, being an important source of a wide range of vital micronutrients (such as beta-carotene, vitamin C, and potassium), phytochemicals, and fiber [1]. The combination of fresh fruits and vegetables with milk is an attractive solution not only to improve sensorial quality of the final products but also the nutritional properties due to the high contents of high biological value proteins as well as water-soluble and fat-soluble vitamins and minerals found in milk.

For these reasons, the World Health Organization (WHO) and the Agencia Española de Seguridad Alimentaria y Nutrición (AESAN) include the consumption of five portions (or 400 g) of fruits and vegetables (excluding starchy roots) per day and a maximum of three lacteous per day for a healthy diet for adults [2,3].

However, these mixtures are susceptible to microbiological and biochemical degradation, so food processing technologies are required to prevent this damage without affecting the freshness. Conventional thermal processing is the most used method; however, it has some disadvantages. Regarding the technological process, thermal processing technologies are less sustainable than non-thermal processing technologies because of water consumption, high costs, and energy. In terms of nutritional and sensorial quality, the thermal process causes the loss of some nutritional and aromatic compounds, change of colour, and the formation of toxic compounds [4,5].

Therefore, several innovative food processing technologies such as ultrasound (USN) or pulsed electric fields (PEF) have been explored due to the great potential that they have alone or in combination with other techniques for preserving minimally processed fresh products [6].

USN technology uses acoustic waves between the 20 kHz and 100 MHz frequency range, which cause the constant growth of gas bubbles in the medium, resulting in bubble collapse and cavitation [7]. High-power USN is applied in the food industry with different objectives, promoting structural modifications in food products, increasing mass transfer, assisting microbial inactivation, or promoting chemical and biochemical reactions, as well as enzyme activation or inhibition, among others. In plant-based foods, USN leads to the disruption of cellular plant tissue, increasing the extractability from the food matrix and the content of bioactive compounds in the medium [8]. This technology is employed in food industry to provide several food matrices, such as meats, fresh products, juices, cereals, and fermented products [9].

PEF has been applied to extend the shelf-life of food and provide safe and higher quality products because of its low temperature and short treatment time. This technology involves the application of electrical treatments of different electric field strength (1–40 kV/cm) for short periods of time (milliseconds) to a product placed between two electrodes [10].

The advantages of this technology are the continuous use, low energy cost, retention of thermolabile compounds (vitamins and bioactive compounds), and no alteration of sensory characteristics. In the industry, it is also used for other purposes, such as improving dehydration processes, reducing the oil absorption capacity of potatoes, and extracting nutrients and bioactive compounds from food matrices and by-products. PEF technology also shows promising results in several food processes, such as enzyme inactivation and promoting both chemical reactions and properties of food macromolecules [11].

Recently, some authors have reported promising applications of USN and PEF in decreasing contaminants (such as pesticides or mycotoxins) [12,13]. The USN mechanisms involved in the release and degradation of food contaminants are based on cavitation, which may result in the generation, growth, and implosion of gas bubbles that will collapse on the surface of the food sample and in the discharge of high pressure and temperature. Both mechanisms could result in the creation of shockwaves and micro-fractures. Concerning PEF, a high voltage increase can induce the vibration and rotation of polar molecules as well as the production of reactive species and radicals. In addition, processing variables such as electric field strength, treatment time, and pulse number can significantly influence the mitigation efficiency [14].

Mycotoxins constitute toxic substances produced by filamentous fungi. *Claviceps*, *Fusarium Aspergillus*, *Alternaria*, and *Penicillium* genus are mainly mycotoxin producers [15]. Mycotoxins are secondary metabolites, i.e., they are not essential molecules for fungal growth and development. They are formed at the end of the exponential phase or at the beginning of the stationary phase of the fungus, and their production depends on environmental conditions. The functions of mycotoxins in fungi are related to differentiation and sporulation. Contamination of crops by toxigenic fungi and, consequently, by mycotoxins can occur during the field, growth, and postharvest (handling and storage) stages. The impact of the presence of mycotoxins on animal productivity, national and international trade, and human health causes significant economic losses [16,17].

A diverse chemical group composition led to several mycotoxin toxic effects, which include nephrotoxicity, genotoxicity, teratogenicity, neurotoxicity, hepatotoxicity, immunotoxicity, membrane damage, gastrointestinal toxicity, cardiotoxicity, pulmonary toxicity, and sometimes a carcinogenic effect. Mycotoxins’ impact on human health depends on many factors, including concentration, method of exposure, and synergistic effect of specific toxins in humans [18]. In this sense, aflatoxins (AFs), *Alternaria* toxins, Ochratoxin A (OTA), and patulin (PAT) are the most common mycotoxins in fruits and fruit-processed foods (such as juices). [19]. The occurrence of these compounds in fruits and their processed products induces severe toxicity at low levels that are hazardous to human health. Moreover, they can also result in economic losses to fruit juice and other food processing establishments [20].

It is after harvest and in the subsequent stages (storage and transport) where fruits, vegetables, and cereals will be more susceptible to attack by fungi, which can subsequently produce mycotoxins and other microorganisms. Post-harvest decontamination methods are all those technologies aimed at the elimination, neutralization, or reduction of mycotoxins present in foods. Therefore, an efficient method for mycotoxin reduction must be able to remove or inactivate mycotoxins from food without producing toxic residues or affecting the technological, nutritional, and sensory properties of the food products. Post-harvest methods do not have a preventive character and will be of great importance in those cases where preventive methods have not been sufficient in controlling the fungus. Post-harvest decontamination methods are classified into biological, chemical, and physical methods [21,22].

Food processing can result in mitigating the risk of mycotoxins to the final consumers, although most mycotoxins are moderately heat-resistant. For instance, baking, frying, roasting, extrusion, and microwave processes have been reported to induce the reduction of mycotoxins in different food matrices [23]. The degree of mycotoxin reduction achieved is highly dependent on processing conditions (temperature, time, water, and pH) as well as being influenced by the matrix, the mycotoxin chemical structure, hydrophobicity, thermal-mechanical susceptibility, and its concentration in the matrix [24]. As mentioned above, in recent years, modern sustainable detoxification methods have emerged as innovative technologies, including ionizing and non-ionizing radiation, cold plasma (C.P.), pulsed light (P.L.), USN, PEF, and High-Pressure Processing (HPP), which are economically and eco-friendly and appropriate, and which maintain the nutritional value and quality of food products. Significant mycotoxin reduction has been reported by several authors. In this regard, PEF technology enhances reduction percentages up to 84% for AFs and in the range 43–70% for emerging mycotoxins in juice samples [12,25]. Concerning USN, its potential has been studied in the removal of aflatoxin B1 (AFB1), deoxynivalenol (DON), zearalenone (ZEA), and OTA in maize and aqueous solution, obtaining degradation rates ranging from 40% to nearly 100% [26,27]. In addition, these authors identified mycotoxin degradation products after the mitigation treatments [25,27]. To the best of our knowledge, scarce information and research is available about the study of the combination effect of various innovative food processing technologies in the mitigation of mycotoxins. However, higher efficiency extraction rates have been reported with the combination of various emerging technologies. For instance, PEF, high-pressure processing, and USN were combined to improve the extraction efficiency. As a result, the combination of PEF and USN could enhanced higher fungi inactivation and mycotoxin degradation rates [28]. The aim of this work is thus to investigate how the sequential combination of USN + PEF and PEF + USN can affect the mycotoxins spiked in an orange juice mixed with milk beverage.

## 2. Materials and Methods

### 2.1. Chemicals and Reagents

Chloroform (CHCl_3_), acetonitrile (ACN), and methanol (MeOH) solvents (99% grade) were provided by Merck (Darmstadt, Germany). Ethyl acetate (EtOAc) (HPLC grade) was supplied from Alfa Aesar (Karlsruhe, Germany). The deionized water with resistivity >18 MΩ cm^−1^ used to prepare the mobile phase was obtained through Milli-Q SP^®^ Reagent Water System (Millipore Corporation, Bedford, MA, USA). In advance of the use, all mobile phase solvents were filtered through a 0.45-µm cellulose filter acquired by Scharlau (Barcelona, Spain). Sodium chloride (NaCl) was bought from VWR Chemicals (Leuven, Belgium), ammonium formate (99%) was purchased from Panreac Quimica S.A.U. (Barcelona, Spain), and formic acid (reagent grade 95%) was obtained from Sigma-Aldrich (St. Louis, MO, USA). Before injection, all samples were filtered through a 13 mm/0.22 µm nylon filter supplied by Membrane Solutions (Plano, TX, USA). OTA and ENNs (Enniatin B (ENNB) and Enniatin B1(ENNB1)) standards were supplied by Sigma and were prepared in methanol at a concentration of 1000 mg/L. Afterwards, working solutions were elaborated from the stock solutions. All solutions were saved at 20 °C.

### 2.2. Sample Preparation

The samples consisting of orange juice mixed with milk were elaborated in the laboratory in accordance with a previous work [29]. First, pectin and sugar were crushed, mixed, and added to hot water previously heated at 50 °C. After 10 min of shaking, hot milk previously heated at 50 °C was added to the solution and shacked until being homogenized. After cooling at room temperature, fresh orange juice and citric acid were added, and the mixture was shacked until being homogenized. Sugar, citric acid, and pectin ingredients were used as a sweetener and a preservative and to give consistency, respectively. The juice composition is contained in the Table 1.

1.5 L was prepared, and three aliquots were taken as untreated controls to test the absence of mycotoxins. 1.2 L was spiked individually by OTA, ENNB and ENNB1 at a concentration of 100 µg/L. Aliquots of 200 mL were then separated to be treated. All experiments were performed in triplicate and all samples were stored at 4 °C until the treatment.

### 2.3. Treatments

**Figure 1:**
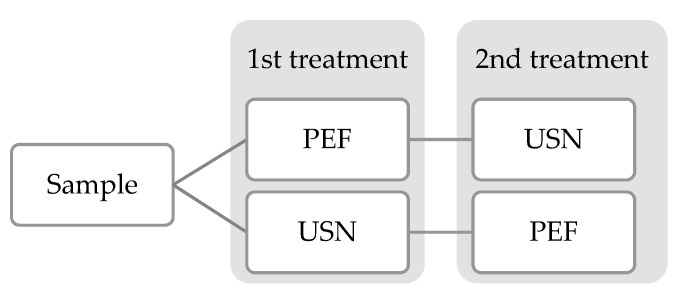
Scheme of the different experimental treatments conducted. PEF: Pulsed Electric Fields, USN: Ultrasounds.

To check the influence of the treatment order (PEF + US vs US + PEF) in the reduction of mycotoxins, the samples were treated following the two following strategies. The first one consisted of using PEF and then USN while the second one applied USN before PEF treatment. The conditions for both treatments were chosen based on the data available in the literature. In previous studies carried out by our research group, PEF treatment allowed significant reduction rates employing a specific energy of 500 kJ/Kg and a voltage of 30 kV, so the same treatment conditions were tested in the present study [12,25]. For the USN treatment, the conditions tested in the literature consisted mainly of treatment times comprising between 10 and 80 min at 20 kHz [13,27], and, therefore, US treatment of 30 min and 20 kHz was tested in the present study in combination with PEF. In both cases, the samples were treated subsequently, and they were stored at 4 °C after treatment (Figure 1).

#### 2.3.1. PEF Treatment

A PEF-Cellcrack III (German Institute of Food Technologies (DIL)) equipment (ELEA, Quakenbrück, Osnabrück, Germany) with a treatment chamber with 10 cm of distance between electrodes was used for the PEF treatment. Treatment parameters were 30 kV of voltage, with a field strength of 3 kV/cm and 500 kJ/kg of specific energy. Approximately 231 pulses were applied in cycles of 20 pulses. Mitigation treatment time was lower than 5 min and temperature did not exceed 75 °C.

#### 2.3.2. USN Treatment

A Branson 5200 ultrasonic bath was employed for the USN treatment. The conditions applied were 20 kHz of frequency and 100 W of power for 30 min at 50 °C.

### 2.4. DLLME

DLLME was the extraction method chosen to extract the mycotoxins from orange juice with milk. [19]. First, 5 mL of the beverage with 1 g of NaCl was agitated for 1 min, and then 950 µL of ACN (dispersant solvent) and 620 µL of EtOAc (extractant solvent) were added and shaken. After this, the mixture was centrifuged at 4000 rpm for 5 min to separate the organic phase (at the top) and it was collected in other tube. A mixture of 950 µL of MeOH (dispersant solvent) and 620 µL of CHCL_3_ (extractant solvent) was then added to the organic phase and a second extraction was carried out. In the same tube, both organic phases were recovered and evaporated. The samples were reconstituted with 1 mL of 20 mM ammonium formate (MeOH/ACN) (50/50 *v*/*v*) and filtered through a 13 mm/0.22 µm nylon filter.

The methodology was validated in a previous study carried out in our research group [19]. In this paper, recovery experiments were performed in a juice matrix at concentrations of 50,100 and 200 µg/L with satisfactory recoveries ranging from 71 to 90% for OTA, between 66–104% for ENB, and ranging from 67 and 110% for ENB1. Regarding matrix effect experiments, SSE (%) of 64 was obtained for OTA and comprised between 54 and 66% for ENNs.

### 2.5. Mycotoxin Determination

Toxins were analyzed by UHPLC-MS/MS using a Sciex TRIPLE QUAD 6500+ mass equipped with electrospray ionization (ESI) coupled to an Agilent 1260 HPLC UHPLC system (degasser, quaternary pump, and column oven) with an Eksigent ULC 100 HTC-xt autosampler. The instrument was equipped with a BEH^®^ C18 Column (1.7 µm 100 Å, LC Column 50 × 2.1 mm, Waters). The mobile phases were (A) H_2_O 5 mM ammonium formate and 0.1% formic acid, and (B) methanol (0.1% formic ac.). The linear gradient was: 0 min (95% A), 2 min (95% A), 13 min (0% A), 15 min (0% A), 15.1 min (5% A), and 18 min (5% A) with an injection volume of 5 µL and a constant temperature of 30 °C in the column. The mass spectrometer was used in positive ionization mode and in multiple Selected Reaction Monitoring (SRM) mode, with a turbo Spray IonDrive ionization source, and with the following conditions: curtain gas (CUR) 30 psi, ion sputtering voltage (IS) at 4.5 kV, temperature of 300 °C, and ion source gas (GS) 1 and 2 at 55 psi. The UHPLC-MS/MS parameters for each compound are listed in Table 2.

### 2.6. Statistical Analysis

Data were analyzed using analysis of variance (one-way ANOVA) to determine the significance of differences between treatments and control samples for each mycotoxin individually. A *p* < 0.05 was considered significant. All analyses were performed with the software GraphPad Prism 8.0.2 (GraphPad Software, San Diego, CA, USA). Data were expressed as mean ± standard deviation in all cases. All analysis performed were applied in triplicate.

## 3. Results

PEF + USN treatment achieved reductions ranging from 34 to 50% while the combination USN + PEF treatment decreased mycotoxin levels from 19 to 37% with respect to the untreated spiked samples. The lowest contents of mycotoxins obtained after PEF + USN treatment were 50.06 ± 15.45 µg/L (OTA) and 52.51 ± 9.04 µg/L (ENNB) and after USN + PEF treatment were also OTA and ENNB (69.78 ± 23.90 and 62.89 ± 12.30 µg/L, respectively).

**Figure 2:**
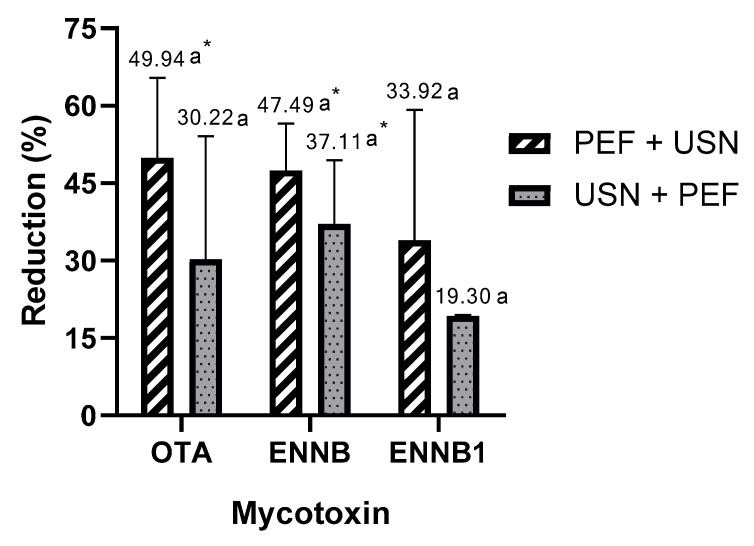
Reduction percentage (%) of Ochratoxin A, Enniatin B, and Enniatin B1 in orange juice with milk after PEF + USN treatment vs. after USN + PEF treatment. (ab, for each mycotoxin different letters indicate different percent reduction rates between treatments) (* indicates a significant reduction concerning the control). OTA: Ochratoxin A, ENNB: Enniatin B, ENNB1: Enniatin B1, USN: Ultrasounds, PEF: Pulsed Electric Fields.

**Figure 3:**
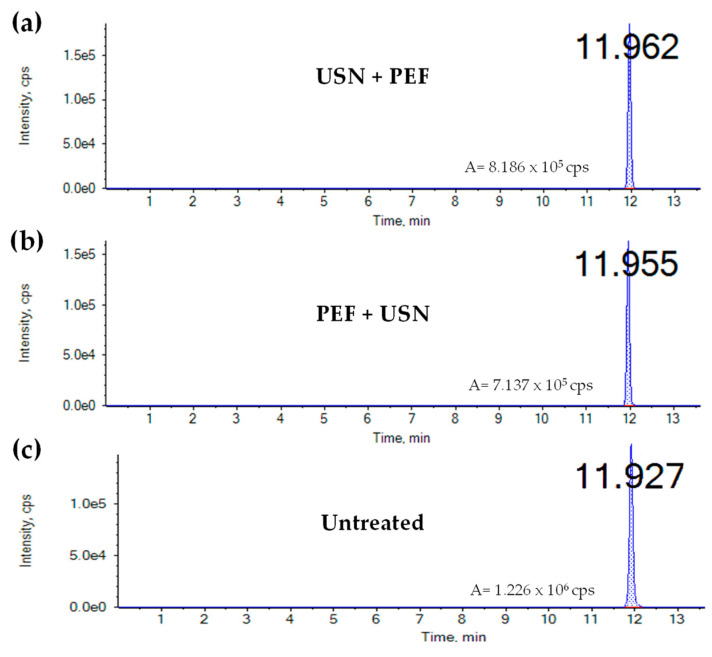
LC-MS/MS-IT chromatograms of orange juice with milk spiked by Enniatin B treated by PEF + USN (**a**) vs. treated by PEF + USN (**b**) vs. untreated (**c**). The area (A) and the retention time (min) also are indicated. USN: Ultrasounds, PEF: Pulsed Electric Fields.

Regarding ENNB1, similar reduction percentages (from 19.30 to 33.92%) were obtained with both combination treatments. Percentage reductions and data contents of mycotoxins after PEF + USN and USN + PEF treatments are listed in Figure 2 and Table 3, respectively. Figure 3 shows the chromatograms of an orange juice milk beverage spiked by ENNB and treated by both combinations and untreated. The highest reduction was obtained for OTA after PEF + USN treatment while ENNB was the mycotoxin with the highest reduction after USN + PEF treatment.

The PEF + USN treatment showed a statistically significant reduction to the control in the juices spiked with OTA and ENNB, and the USN + PEF treatment allowed a significant reduction in juices spiked with ENNB. For the three mycotoxins studied, no significant differences were observed in the samples treated by PEF + USN and USN + PEF.

## 4. Discussion

Regarding the studies found in the available literature about PEF effect in mycotoxin content, Pallarés et al. [12] studied the ENNs reduction in juice and smoothies treated by PEF. The content of ENNA, ENNA1, ENNB, and ENNB1 in juices and smoothies was reduced by 50% approximately after applying PEF treatment, with the same conditions as in this study (Table 4). ENNB and ENNB1 were reduced by 43% in grape juice, while in smoothies, ENNB and ENNB1 were reduced by 57% and 60%, respectively. In the present study, ENNB1 achieved similar percent reductions after both treatments to those reported by Pallarés et al. [30], although lower reductions were found for ENNB1. This difference in reduction percentages could be due to the different interaction of ENNB1 with the food matrix [31,32].

In another study, Pallarés et al. [25] also reported the effect of the PEF treatment in grape juice on AFs. Aflatoxin B2 (AFB2) and aflatoxin G2 (AFG2) were reduced up to 84% while AFB1 and AFG2 were reduced by less than 30%. In the same study, grape juice was also treated with HPP, and the reduction was lower than 30%. Subramanian et al. [33] and Vijayalakshmi et al. [34] obtained higher reduction percentages, between 78 to 96%, for AFs treated by PEF in combination with heat and pH in potato dextrose agar.

Moreover, in juice matrix, the effect of HPP technology has been studied. For instance, Avsaroglu et al. [32] and Hao et al. [31] used HPP to decrease PAT in apple juice. These authors applied 400 MPa for 5 min in combination with 50 °C and used 600 MPa for 5 min, respectively. Both studies obtained a PAT reduction of 30%, and so both studies are in close agreement.

In another study, Pallarés et al. [30] studied the ENNs content in different juice formulations after HPP treatment. In this study, juices were processed at 600 MPa for 5 min. The reduction percentages ranged from 11 to 75% and ENNA1 was the only mycotoxin reduced in all formulations (orange, strawberry juice with milk, and grape juice). ENNB and ENNB1 were reduced in orange juice by 37 and 23%, respectively. These results agree with the reductions obtained by PEF in this study.

USN has been applied to reduce mycotoxin levels, but, to the best of our knowledge, no information is available about its possible applications in juice matrix decontamination. Nevertheless, in fruits (dried fig), the pre-drying treatments of K_2_CO_2_ emulsion in combination with USN enhanced the fungal growth control and, in consequence, the mycotoxin production. [35]. For instance, in the maize and aqueous solution, Liu et al., [13] observed high degradation levels for AFB1, DON, ZEA, and OTA (96.5, 60.8, 95.9 and 91.6%, respectively). These authors also reported that the mycotoxin degradation was significantly affected by the ultrasonic intensity (2.2–11 W/cm^3^) and sonication time range (from 10 to 50 min). In addition, they observed AFB1 degradation rate up to 85.1% in aqueous solution at frequency of 20 kHz, power intensity of 6.6 W/cm^3^, and 80 min of treatment [27]. In contrast, Mortazavia, Sania, and Mohsenib [26] reported lower reductions (~41%) for AFs after 10 min at 20 KHz of frequency. In the present study, the reductions obtained after treatments of 30 min at 30 kH were up to 50%. The different reduction rates observed could be attributed to the treatment duration. 

Several authors have evaluated the stability of ENNs after thermal processing in different food matrices, such as pasta, fish, and medicinal plants. ENNs are not thermostable, and, therefore, during the cooking process (100 °C, 5 or 10 min), the ENNs are reduced up to 100% [36]. Although at high temperatures the ENNs are totally reduced, this also causes the loss of the nutritional and organoleptic quality of the juices and smoothies [6,37]

The OTA thermostability has also been studied. For instance, Dahal et al. [38] observed a high thermostability of OTA in water heated at 100 °C. Moreover, Lee [39] studied OTA reduction in roasted oat-based cereals, observing an OTA reduction lower than 2% after at 120 °C 30 min. In our study, both treatments showed higher OTA reductions in a shorter treatment length of time.

**Table 4:** Summary of literature available on the reduction percentages of mycotoxins in food products after the emerging treatments.

Treatment	Type of Matrix	Mycotoxin	Treatment Conditions	% Reductions Achieved	Reference
PEF	Potato dextrose agar	AFs	Frequency 50 Hz, burst 10, energy 1 kJ, time 10 s (+ heat)	79 to 96	[33]
	Model system	AFs	Frequency 50 Hz, burst 10, energy 1 kJ, time 10 s (+ pH 4 to 10)	77 to 97	[34]
	Juice and smoothie	ENNs and BEA	Voltage 30 kV, field strength 3 kV/cm, specific energy 500 kJ/kg	43 to 70	[12]
	Grape juice	AFs	Voltage 30 kV, field strength 3 kV/cm, specific energy 500 kJ/kg	22 to 84	[25]
USN	Aqueous solution	AFB1, DON, ZEA, OTA	Frequency 20 kHz, power intensity 11 W/cm^3^, time 50 min	61 to 97	[13]
	Aqueous solution	AFB1	Frequency 20 kHz, power intensity 6.6 W/cm^3^, time 80 min	85.1	[27]
	Aqueous solution	AFs	Frequency 20 kHz, power output 1000 W, time 10 min	41	[26]
HPP	Orange juice	AFB1	Pressure 600 MPa, time 5 min	24 29	[40]
		AOH			
	Juice	ENNs	Pressure 600 MPa, time 5 min	11 to 75	[30]
	Grape juice	AFs	Pressure 500 MPa, time 5 min	14 to 29	[25]
	Vegetable juices	PAT	Pressure 600 MPa, time 5 min	30	[31]
	Apple Juice	PAT	Pressure 400 MPa, time 5 min, (+ heat)	29	[32]
Combination of PEF and USN	Orange juice with milk	OTA, ENNB, ENNB1	USN: Frequency 20 kHz, power 100 W, time 30 min PEF: Voltage 30 kV, field strength 3 kV/cm, Specific energy 500 kJ/kg	up to 50% for OTA (PEF + USN) up to 47% for ENNs (PEF + USN)	This study

PEF: Pulsed Electric Fields, USN: Ultrasounds, HPP: High-Pressure Processing, AF: Aflatoxin, AFB1: Aflatoxin B1, ENN, Enniatin, BEA: Beauvericin, DON: Deoxynivalenol, ZEA: Zearalenone, OTA: Ochratoxin A, AOH: Alternariol, PAT: Patulin. ENNB: enniatin B, ENNB1: Enniatin B1, ENNs: enniatins.

## 5. Conclusions

PEF and USN treatments are effective technologies to mitigate ENNs and OTA in juices beverages. The combination of PEF and USN promoted ENNs reduction between 19% to 47%, while OTA reduction ranged from 30 to 50% in orange juice with milk. After PEF + USN treatment, the highest OTA reduction (50%) was found, while the most significant ENNB reduction (47%) was found after the combined application of PEF + USN treatment. ENNB1 also obtained higher percentages of reductions after PEF + USN treatment (34%). No statistically significant differences have been observed in the order of the treatments, but the combination of PEF + USN showed higher reduction percentages, and, therefore, more studies are needed to clarify this point. Despite the benefits observed by the pulsed electrical pulse method in combination with USN technology in the mitigation of mycotoxins, more studies are needed on its efficacy in other food matrices, and more studies are also needed to establish the mechanisms by which this phenomenon of mitigation occurs.

## Figures and Tables

**Table 1:** Orange juice with milk ingredients per 100 mL.

Orange Juice	Milk	Water	Pectin	Sugar	Citric Acid
50 mL	20 mL	30 mL	0.3 g	7.5 g	0.1 g

**Table 2:** Optimized mass spectrometry UHPLC-MS/MS parameters for the determination of Ochratoxin A, Enniatin B, and Enniatin B1.

Mycotoxin	*Precursor Ion (m*/*z)*	*Quantifier Product Ion (m*/*z)*	*Qualifier* *Product* *Ion (m*/*z)*	*t*_R_ (min)	DP	EP	CE	CXP
OTA	404	239	102	101	91	10	37	16
ENNB	6575	1963	2141	12	81	10	45	18
ENNB1	6714	196	210	12,2	111	10	43	12

*t*_R_: retention time, DP: declustering potential, EP: entrance potential, CE: collision energy, CXP: collision cell exit potential, OTA: Ochratoxin A, ENNB: Enniatin B, ENNB1: Enniatin B1.

**Table 3:** Contents of Ochratoxin A, Enniatin B, and Enniatin B1 in orange juice with milk after the combination of treatments selected.

	OTA	ENNB	ENNB1
PEF + USN	50.06 ± 15.45 µg/L	52.51 ± 9.04 µg/L	66.08 ± 25.29 µg/L
USN + PEF	69.78 ± 23.90 µg/L	62.89 ± 12.30 µg/L	80.70 ± 0.20 µg/L

OTA: Ochratoxin A, ENNB: Enniatin B, ENNB1: Enniatin B1. The contents of non-treated samples were approximately 100 µg/L (the initial spiked level).

## Data Availability

Data is contained within the article.
